# Propionate Ameliorates Dextran Sodium Sulfate-Induced Colitis by Improving Intestinal Barrier Function and Reducing Inflammation and Oxidative Stress

**DOI:** 10.3389/fphar.2016.00253

**Published:** 2016-08-15

**Authors:** Ling-chang Tong, Yue Wang, Zhi-bin Wang, Wei-ye Liu, Sheng Sun, Ling Li, Ding-feng Su, Li-chao Zhang

**Affiliations:** ^1^Department of Pharmacy, Shanghai Municipal Hospital of Traditional Chinese MedicineShanghai, China; ^2^Department of Pharmacology, College of Pharmacy, Second Military Medical UniversityShanghai, China; ^3^Department of Pharmacy, Ningxia Medical UniversityYinchuan, China

**Keywords:** ulcerative colitis, short-chain fatty acid, propionate, intestinal barrier function, tight junction protein, inflammation, dextran sulfate sodium

## Abstract

Propionate is a short chain fatty acid that is abundant as butyrate in the gut and blood. However, propionate has not been studied as extensively as butyrate in the treatment of colitis. The present study was to investigate the effects of sodium propionate on intestinal barrier function, inflammation and oxidative stress in dextran sulfate sodium (DSS)-induced colitis mice. Animals in DSS group received drinking water from 1 to 6 days and DSS [3% (w/v) dissolved in double distilled water] instead of drinking water from 7 to 14 days. Animals in DSS+propionate (DSS+Prop) group were given 1% sodium propionate for 14 consecutive days and supplemented with 3% DSS solution on day 7–14. Intestinal barrier function, proinflammatory factors, oxidative stress, and signal transducer and activator of transcription 3 (STAT3) signaling pathway in the colon were determined. It was found that sodium propionate ameliorated body weight loss, colon-length shortening and colonic damage in colitis mice. Sodium propionate significantly inhibited the increase of FITC-dextran in serum and the decrease of zonula occludens-1 (ZO-1), occludin, and E-cadherin expression in the colonic tissue. It also inhibited the expression of interleukin (IL)-1β, IL-6, and tumor necrosis factor-α (TNF-α) mRNA and phosphorylation of STAT3 in colitis mice markedly, reduced the myeloperoxidase (MPO) level, and increased the superoxide dismutase and catalase level in colon and serum compared with DSS group. Sodium propionate inhibited macrophages with CD68 marker infiltration into the colonic mucosa of colitis mice. These results suggest that oral administration of sodium propionate could ameliorate DSS-induced colitis mainly by improving intestinal barrier function and reducing inflammation and oxidative stress via the STAT3 signaling pathway.

## Introduction

Inflammatory bowel disease (IBD), including Crohn’s disease and ulcerative colitis, is a group of chronic inflammatory disorders of the gastrointestinal tract characterized by intestinal inflammation and mucosal damage (Quetglas et al., 2015). It is commonly believed that intestinal barrier function destruction, intestinal flora disturbance, and immune dysfunction play important roles in the pathogenesis of IBD (Quetglas et al., 2015; Loddo and Romano, 2015). Glucocorticoids, sulfasalazine, and immunosuppressive drugs have been traditionally used for the treatment and maintenance of ulcerative colitis. However, clinical application of these drugs is limited by their adverse effects (Mao and Hu, 2016), and therefore there is an urgent need to seek alternative remedies.

Increasing the intake of fermentable dietary fibers or short-chain fatty acids (SCFAs) seems to be clinically beneficial to the treatment of colitis (Cabre and Domenech, 2012). SCFAs, predominantly acetate, propionate, and butyrate, are produced in the colonic lumen by anaerobic fermentation of undigested carbohydrates, crude fibers, and polysaccharides (Bolognini et al., 2016). Depending on diet and gut microbiota composition, the intestinal SCFA concentration can range from 60 to 150 mmol/L (Hill, 1995), with butyrate, propionate, and acetate in a nearly constant molar ratio of 15:25:60, respectively (D’Argenio and Mazzacca, 1999). The physiological effects of SCFAs have been well documented, which include reducing the production of proinflammatory factors (Huang and Wu, 1997; Meijer et al., 2010), enhancing intestinal barrier function (Mariadason et al., 1997, 1999; Peng et al., 2007; Suzuki et al., 2008; Van Deun et al., 2008; Elamin et al., 2013), inhibiting oxidative stress (Hamer et al., 2009, 2010), and preventing colon carcinogenesis (Clausen et al., 1991; Hijova and Chmelarova, 2007) *in vitro, in vivo*, and in animals. However, most previous studies mainly focused on butyrate, and few studies have devoted their efforts to other SCFAs such as propionate, although it is abundant as butyrate in the gut and blood.

The aim of the present study was to investigate the effects of sodium propionate on intestinal barrier function and the expression of tight junction protein in mice with colitis induced by dextran sulfate sodium (DSS). In addition, the effects of sodium propionate on inflammation and oxidative stress and signal transducer and activator of transcription 3 (STAT3) signaling pathway were also investigated.

## Materials and Methods

### Materials

The main materials used in this study were DSS (molecular weight 36–50 kDa, MP Biomedicals, Inc., Aurora, OH, USA); sodium propionate (Sinopharm Chemical Reagent CO., Ltd., Shanghai, China); Trizol reagent (Invitrogen, Carlsbad, CA, USA); PrimeScript RT Master Mix Perfect Real Time kit (Takara Biotechnology, Dalian, China); FastStart Universal SYBR Green Master (Rox; Roche, Mannheim, Germany); superoxide dismutase (SOD), myeloperoxidase (MPO), and catalase (CAT) kit (Jiancheng, Nanjing, China).

The main reagents used in this study were antibodies for E-cadherin and occluding (Cell Signaling Technology, Danvers, MA, USA; Life Technologies Inc., Gaithersburg, MD, USA); zonula occludens-1 (ZO-1) antibody and β-actin (Santa Cruz Biotechnology, Inc., Santa Cruz, CA, USA; Sigma–Aldrich, St. Louis, MO, USA); mouse anti-CD68 monoclonal antibody (Abcam, Cambridge, England); mouse anti-STAT-3 monoclonal (124H6) and anti-p-STAT-3 (Tyr705) antibodies (Cell Signaling Technology, Danvers, MA, USA).

### Animals

C57BL/6J male mice weighing 18–22 g were obtained from SLRC Laboratory Animal Lid (Shanghai, China). All mice were kept under an automated 12 h/12 h dark-light cycle at a controlled temperature of 22°C ± 2°C, relative humidity of 50–60% and allowed free access to standard dry diet and tap water *ad libitum*. All animals received humane care, and experimental procedures were performed in accordance with the guidelines of the Second Military Medical University for health and care of experimental animals.

### Dextran Sodium Sulfate-Induced Colitis

Forty mice were equally randomized to four groups: a control group, a propionate group, a DSS group, and a DSS+propionate (DSS+Prop) group. Mice in control and propionate groups received drinking water and sodium propionate [1% (w/v) dissolved in double distilled water], respectively, for 14 consecutive days. Mice in DSS group received drinking water from 1 to 6 days and DSS [3% (w/v) dissolved in double distilled water] instead of drinking water from 7 to 14 days. Mice in DSS+prop group received sodium propionate [1% (w/v)] from 1 to 6 days plus DSS [1% (w/v)] from 7 to 14 days. The disease activity index including weight loss, stool consistency, and fecal blood was evaluated every day. At day 15, all animals were sacrificed by dislocation of the cervical vertebra for blood and organ collection.

### Histopathological Assessment

All mice were sacrificed for histological assessment. The colon length was first measured for each group, and then the colon was cut into segments for further detection. Colons in the same position were fixed in 4% neutral formalin, paraffin embedded, and HE stained routinely. Histopathological changes were evaluated by using the histological injury scale as described previously (Wirtz et al., 2007). The criteria for evaluation were as follows: 0: no obvious inflammatory reaction; 1: the presence of low-level inflammatory reaction with a few scattered inflammatory cells; 2: the presence of moderate inflammatory infiltration; 3: the presence of severe inflammatory reaction in the colon tissue as represented by increased vascular density and thickness; 4: the presence of large amounts of inflammation cell infiltration and rupture of goblet cell mass.

### *In vivo* Intestinal Permeability

The *in vivo* intestinal epithelial permeability was determined as described previously (Moussaoui et al., 2014). Briefly, mice were fasted overnight and FITC-dextran solution (4 kDa, 600 mg/kg) was delivered via gavage. Mice were sacrificed at 4 h after intragastric administration, and blood was harvested via cardiac puncture and then separated by centrifugation. Serum levels of FITC were read at 480 and 520 nm on a microplate fluorometer.

### Protein Extraction and Immunoblotting Analysis

Immunoblotting was performed as described previously (Zhang et al., 2011). Briefly, the colon tissue was sliced into sections and washed with PBS. Tissue proteins were extracted by lysing in RIPA buffer containing protease and phosphatase inhibitor cocktail. The mixture was centrifuged at 14,000 × *g* and 4°C for 15 min, and the protein content in the supernatant was determined by Bradford method. An equal amount of protein was separated by 10% (w/v) sodium dodecyl sulfate-polyacrylamide gel electrophoresis (SDS-PAGE) and transferred to nitrocellulose membranes. The membranes were blocked for 3 h at room temperature with blocking reagent, and the primary antibodies were incubated overnight at 4°C. The antibodies included anti-ZO-1 (1:1000), anti-E-cadherin (1:1000), anti-occludin antibody (1:2000), anti-STAT3 (1:1000), anti-p-STAT3 (1:1000), and anti-β-actin antibody (1:5000). After washing with PBST, the membranes were incubated with corresponding secondary antibodies (1:10000 dilution) for 50 min at room temperature. Specific bands were scanned and analyzed by Odyssey Infrared Imaging System (LI-COR, Lincoln, NE, USA). β-actin was used as the protein loading control. All immunoblotting experiments were repeated at least three times.

### RNA Isolation and Quantitative RT-PCR

Total RNA was extracted from the colon tissue in each group with Trizol reagent according to the manufacturer’s instructions. After reverse transcription, complementary DNA was used as templates for PCR. Primers for the inflammatory factors and internal reference were as follows: tumor necrosis factor-α (TNF-α): forward, 5′-CATTTCCACG ATTTCCCAGA-3′, reve-rse, 5′-GGAAAGCCCATTTGAGTCCT-3′; interleukin (IL)-1β: forward, 5′-CTCACAAGCAGAGCACAAGC-3′, reverse, 5′-CAGTCCAGCCCATA CTTTAGG-3′; IL-6: forward, 5′-CGGAGAGGAGACTTCACAGAG-3′, reverse, 5′-CATTTCCACGATTTCCCAGA-3′; GAPDH: forward, 5′-GTATGACTCCACTC ACGGCAAA-3′, reverse, 5′-GGTCTCGCTCCTGGAAGATG-3′. The housekeeping gene GAPDH was used as internal control, and the amount of RNA was calculated by the comparative threshold cycle method as recommended by the manufacturer. Quantitative real-time PCR was carried out by ABI 7500 real-time PCR system (Applied Biosystems, Foster, CA, USA).

### Measurement of Myeloperoxidase (MPO) Level in Colon and Serum

The ability of MPO to modulate the hydrogen peroxide level was used to measure MPO activity by using a modified method according to the manufacturer’s instructions. The freshly excised colon was rinsed, homogenized in tissue lysis buffer, and then centrifuged. Pellets were re-suspended in PBS containing 0.5% hexadecyl-trimethylammonium bromide and then freeze-thawed three times. Absorbance was recorded at 460 nm. The protein concentration was determined using the BCA-100 Protein Determination Kit (Bocai Biotechnology, Shanghai, China). Finally, MPO activity was defined as the quantity of enzyme degrading 1 μmol/ml of peroxide at 37°C, expressed in unit/mg protein.

### Detection of CAT and SOD Level in Colon and Serum

The freshly excised colon was rinsed, homogenized in tissue lysis buffer, and then centrifuged. CAT and SOD activities in tissue lysate and serum were measured by using the CAT or SOD kit according to the manufacturer’s instruction (Nanjing Jiancheng Bioengineering Institute, Nanjing, China). Using a microplate fluorometer, CAT and SOD activities were measured at 405 and 450 nm, respectively. The protein concentration was measured by Bradford method. The concentration of CAT and SOD in colon was presented as picograms per milligram colon protein.

### Assessment of Macrophages in Colonic Mucosa by Immunofluorescence

Immunofluorescence was performed as described previously (Liu et al., 2015). The 5 μm paraffin-embedded colonic tissue sections were de-paraffinized in xylene and then rehydrated in ethanol solution. The slides were blocked with 5% BSA in TBS for 90 min. The sections were incubated with anti-CD68 antibody at a dilution of 1:100 overnight at 4°C. After the sections were washed three times with TBS, the slides were incubated with Alexa Fluor 488 secondary antibody diluted 1:200 with TBS and incubated in the dark for 120 min at room temperature. The sections were mounted with mounting medium containing 4,6-diamidino-2-phenylindole (DAPI; Beyotime Institute of Biotechnology, Shanghai, China) for nuclear counterstaining and visualized under a fluorescent microscope (Olympus IX71, Tokyo, Japan). Macrophages were counted per square millimeter (mm^2^) at a magnification of 400× using a grid ocular. Only cells containing a nucleus stained by DAPI were considered. Counting was performed by two observers independently who were blind to the diagnosis of the specimen. The difference of their results was never greater than 10%, and the mean value was used.

### Statistical Analysis

All quantified data were expressed as means ± SD. Data involving more than two groups were assessed by analysis of variance (ANOVA). Values of *P* < 0.05 were considered statistically significant. Statistical analysis was performed using SPSS (Version 18.0 for Windows, SPSS Inc., Chicago, IL, USA).

## Results

### Effects of Sodium Propionate on Body Weight, Colon Length, and Histopathology in DSS-Induced Colitis Mice

There was no significant difference in body weight, colon length, histological evaluation, and histological score between control and propionate groups.

**FIGURE 1:**
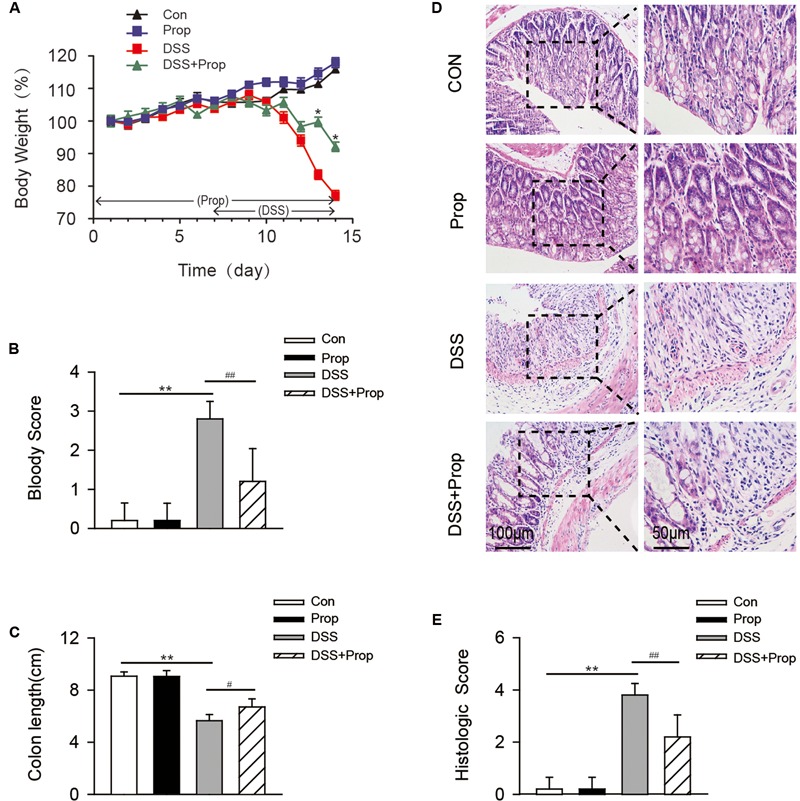
**Effects of sodium propionate on body weight, bloody score, colon length, and histopathology in DSS-induced colitis mice.** Mice were divided into four groups. Animals in control and propionate groups received water and sodium propionate [1% (w/v) dissolved in water] alone, respectively, during the 14-day treatment period. Animals in DSS group received drinking water from 1 to 6 days and DSS [3% (w/v) dissolved in double distilled water] instead of drinking water from 7 to 14 days. Animals in DSS+propionate (DSS+Prop) group were given 1% sodium propionate for 14 consecutive days and supplemented with 3% DSS solution on day 7–14. Body weight **(A)**, bloody score **(B)**, and colon length **(C)** were examined. HE stained colon tissue sections were analyzed for histopathology **(D)**. Images were representative of 6–8 mice. Colon injury scores were also determined **(E)**. Scar bars were 100 and 50 μm, respectively. Data are presented as mean ± SD, *n* = 10 per group. ^∗^*P* < 0.05, ^∗∗^*P* < 0.01 vs. control group; ^#^*P* < 0.05, ^##^*P* < 0.01 vs. DSS group.

Compared with control group, mice treated with DSS showed body weight loss on day 13 and day 14 (**Figure 1A**). However, the administration of sodium propionate significantly improved the body weight loss on day 13 and day 14 as compared with DSS group (19.56 ± 1.43 vs. 16.40 ± 0.44, 18.06 ± 1.40 vs. 15.14 ± 0.50, respectively; **Figure 1A**).

Compared with control group, the colon length was significantly shortened in DSS-induced mice.

Compared with DSS group, the administration of sodium propionate significantly increased the colonic length (6.70 ± 0.62 vs. 5.64 ± 0.48, respectively; **Figure 1C**).

Dextran sulfate sodium-induced mice presented more serious intestinal bleeding than mice in control group.

Compared with DSS group, sodium propionate ameliorated the intestinal bleeding (1.20 ± 0.84 vs. 2.80 ± 0.45, respectively; **Figure 1B**).

The histological and morphological characteristics of the colon were assessed by HE staining. The colon from control group showed intact morphology and substantial goblet cells. However, the colon from DSS group presented serious ulcers in the colon membrane, and the number of goblet cells was decreased obviously, causing a high histological score. Compared with DSS group, sodium propionate ameliorated the intestinal ulcer, and blocked neutrophil cell infiltration with minimal loss of goblet cells, resulting in a low histological damage score (2.20 ± 0.84 vs. 3.80 ± 0.45, respectively; **Figures 1D,E**). These results suggest that sodium propionate could ameliorate tissue injury induced by DSS.

### Sodium Propionate Improves Intestinal Barrier Function in DSS-Induced Colitis Mice

**FIGURE 2:**
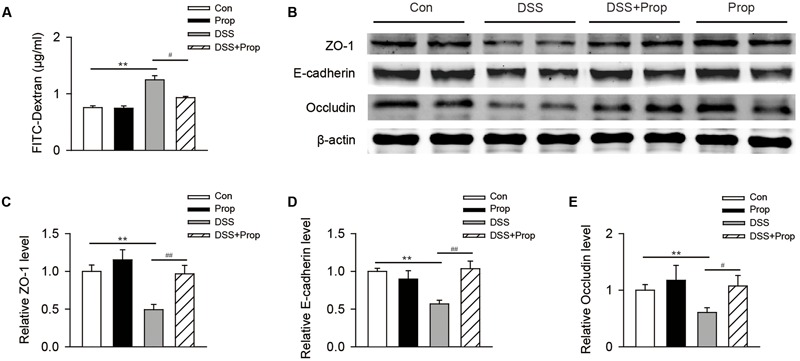
**The effects of sodium propionate on intestinal barrier function in DSS-induced mice.** The FITC-dextran levels in serum were determined **(A)**. *n* = 10 per group. The expression of tight junction and associated protein ZO-1, E-cadherin and occluding in colonic tissues was determined by immunoblotting **(B–E)**. β-actin was used as the protein loading control. *n* = 4 per group. Representative blots of three independent experiments are shown. Data are presented as mean ± SD. ^∗^*P* < 0.05, ^∗∗^*P* < 0.01 vs. control group; ^#^*P* < 0.05, ^##^*P* < 0.01 vs. DSS group.

Intestinal barrier function plays an important role in maintaining normal bowel function by preventing harmful substances such as intestinal bacteria and toxins from going into other tissues or blood circulation via the intestinal mucosa. The destruction of the intestinal barrier function would lead to colitis and even systemic inflammatory response syndrome (Colgan et al., 2015). There was no significant difference in serum FITC-dextran and the expression of tight junction associated proteins between control and propionate groups. Serum FITC-dextran in DSS-induced colitis mice was higher than that in control mice. However, sodium propionate significantly inhibited the increase of FITC-dextran in serum (0.93 ± 0.02 vs. 1.25 ± 0.07, respectively; **Figure 2A**). Compared with control group, the expression of tight junction associated proteins including ZO-1, E-cadherin, and occludin, was decreased in DSS-induced colitis mice, indicating that intestinal barrier function was damaged seriously. Compared with DSS group, sodium propionate significantly increased the level of TJ associated proteins include ZO-1 (0.97 ± 0.11 vs. 0.49 ± 0.07, respectively), E-cadherin (1.03 ± 0.10 vs. 0.57 ± 0.05, respectively) and occludin (1.07 ± 0.18 vs. 0.61 ± 0.08, respectively; **Figures 2B–E**), indicating that sodium propionate might contribute to the protection of intestinal barrier function.

### Sodium Propionate Attenuates the Trend of Increased Expression of Proinflammatory Factor mRNA and Inhibits the Activation of STAT3 Signaling Pathway

There was no significant difference in mRNA expression level of proinflammatory factors and STAT3 between control and propionate groups.

**FIGURE 3:**
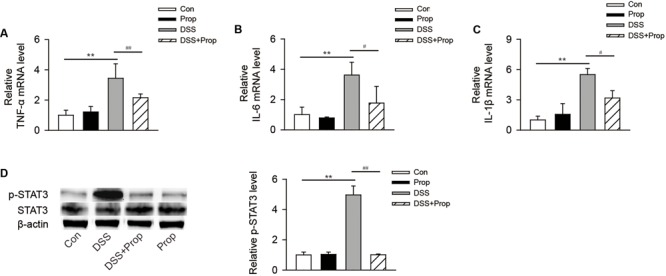
**The effects of sodium propionate on the expression of proinflammatory factor mRNA and STAT3 phosphorylation in colitis mice.** The expression level of proinflammatory factor mRNA was detected by real-time PCR. The mRNA level in each group was determined by relating to the level in control group (defined as 100%; **A–C**). STAT3 expression in the colon was determined by immunoblotting **(D)**. β-actin was used as the protein loading control. *n* = 4 per group. Representative blots of three independent experiments are shown. ^∗^*P* < 0.05, ^∗∗^*P* < 0.01 vs. control group; ^#^*P* < 0.05, ^##^*P* < 0.01 vs. DSS group.

Compared with control mice, the mRNA expression levels of proinflammatory factors such as TNF-α, IL-1β, and IL-6 were increased markedly. Compared with DSS group, sodium propionate significantly inhibited the expression of TNF-α (2.15 ± 0.25 vs. 3.43 ± 0.96, respectively), IL-1β (3.15 ± 0.69 vs. 5.50 ± 0.60, respectively), and IL-6 (1.77 ± 1.09 vs. 3.62 ± 0.84, respectively; **Figures 3A–C**).

Compared with control group, the phosphorylation level of STAT3 was increased in DSS-induced colitis mice. However, sodium propionate significantly inhibited the phosphorylation of STAT3 and decreased inflammatory reaction accordingly (1.00 ± 0.05 vs. 4.96 ± 0.59, respectively; **Figure 3D**).

### Sodium Propionate Inhibits Oxidative Stress in DSS-Induced Colitis Mice

**FIGURE 4:**
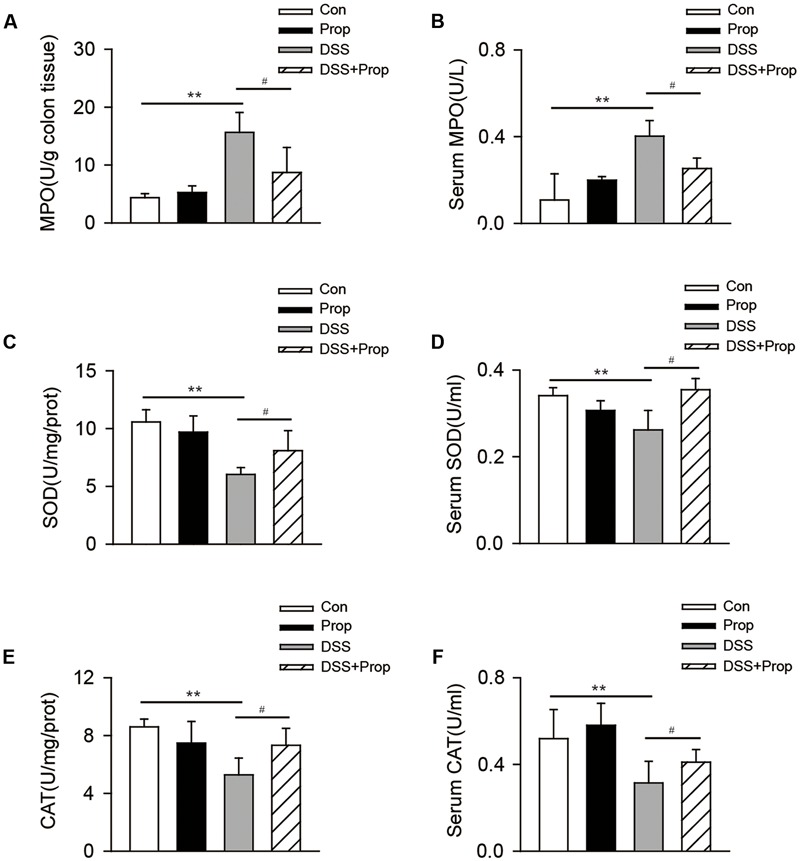
**The effects of sodium propionate on oxidative stress response in colitis mice.** MPO, SOD, and CAT levels in colonic tissues were determined **(A,C,E)**. MPO, SOD, and CAT levels in serum were shown **(B,D,F)**. Data are presented as mean ± SD. *n* = 10 per group, ^∗^*P* < 0.05, ^∗∗^*P* < 0.01 vs. control group; ^#^*P* < 0.05, ^##^*P* < 0.01 vs. DSS group.

Oxidative stress is considered an important factor in colitis in that it can induce the expression of oxygen free radical and lead to fat, protein, and DNA damage (Balmus et al., 2016). MPO, known as a promoting agent for oxidative stress, was significantly increased in serum and colonic tissue in DSS-induced mice compared with control mice. Sodium propionate decreased the level of MPO in colon (8.69 ± 4.30 vs. 15.61 ± 3.45, respectively) and serum (0.25 ± 0.05 vs. 0.40 ± 0.07, respectively) compared with DSS group (**Figures 4A,B**).

Accordingly, the levels of anti-oxidative stress factors including SOD and CAT in serum and colon were significantly decreased in DSS-induced mice compared with the control. Compared with DSS group, sodium propionate increased the level of SOD in colon (8.13 ± 1.56 vs. 6.05 ± 0.46, respectively) and in serum (0.35 ± 0.03 vs. 0.26 ± 0.04, respectively); meantime, sodium propionate increased the level of CAT in colon (7.33 ± 1.16 vs. 5.27 ± 1.16, respectively) and in serum (0.41 ± 0.06 vs. 0.31 ± 0.10, respectively) (**Figures 4C–F**). There was no significant difference in serum and colon levels of MPO, SOD, and CAT between control and propionate groups.

### Sodium Propionate Inhibits Expression of CD68 in DSS-Induced Colitis Mice

**FIGURE 5:**
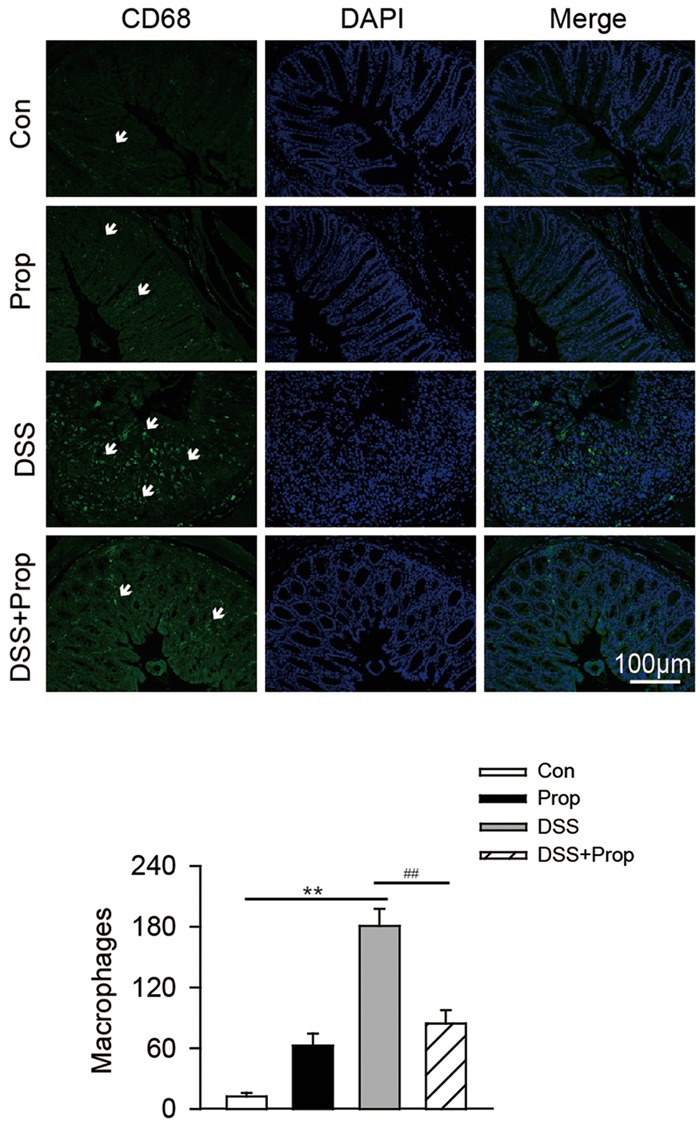
**The effects of sodium propionate on macrophages with CD68 marker in the colonic tissue.** Macrophages were determined by immunofluorescence in colonic tissues. CD68 was used as the marker of macrophage/monocyte infiltration in the colon of mice and visualized by fluorescence microcopy (green staining). Nuclei were stained with DAPI (blue staining). Arrows indicate CD68-labeled macrophages in the colon. Images were representative of 6–8 mice in each group. Scar bars were 100 μm. Morphometric analysis of macrophages in the colon was also performed. Data are presented as mean ± SD. *n* = 6–8 per group, ^∗^*P* < 0.05, ^∗∗^*P* < 0.01 vs. control group; ^#^*P* < 0.05, ^##^*P* < 0.01 vs. DSS group.

Destruction of the intestinal barrier can lead to macrophage infiltration into the colon, which can exacerbate the inflammatory process. Expression of CD68 serves as a marker of macrophages/monocytes in the colon. There was no significant difference in CD68 expression between control and propionate groups. The expression of CD68 was significantly increased in DSS-induced mice compared with the control. However, sodium propionate reduced the expression of CD68 in the colon compared with DSS-induced colitis mice. Quantification of CD68 positive macrophages also showed same result (84.33 ± 13.05 vs. 180.67 ± 16.65, respectively; **Figure 5**).

## Discussion

In the present study, we found that sodium propionate inhibited the down-regulation of tight junction proteins such as ZO-1, occludin, and E-cadherin, and improved the impaired intestinal barrier function induced by DSS. Sodium propionate also reduced the expression of pro-inflammatory factors TNF-α, IL-1β, and IL-6 mRNA in colon tissues. Moreover, sodium propionate inhibited oxidative stress in the colon by reducing MPO activity and enhancing SOD and CAT activities in serum and colon.

Intestinal epithelial barrier defects have been recognized as an important pathogenic factor in a number of inflammatory conditions of the gut, including CD and UC (Sánchez de Medina et al., 2014). Intestinal epithelial barrier defects are characterized by increased intestinal permeability. Tight junctions and adherence junctions mainly restrict and modulate intestinal permeability (Lee, 2015). In this study, we chose occludin and E-cadherin as the representative proteins of tight junctions and adherence junctions, and used ZO-1 as the representative protein of connecting transmembrane proteins to cytoskeleton proteins. It was found that sodium propionate increased the serum FITC-dextran level and inhibited the down-regulation of tight junctions and its associated protein occludin, E-cadherin, and ZO-1 in the colon of colitis mice. A recent study showed that multi-fiber mix feeding increased the concentration of total SCFA, acetate, propionate and butyrate in the caecum and epithelial expression and correct localization of tight junction proteins (occludin and ZO-1) in IL-10^-/-^ mice (Wang et al., 2016). A more previous study also showed that the amount of dietary fiber significantly altered the barrier function by reducing paracellular permeability in the distal colon of normal rats (Mariadason et al., 1999). It was found that applying the SCFA mixture to the intestinal mucosa of anesthetized rats suppressed [^3^H] mannitol transport from the caecal lumen to the mesenteric blood in a dose-dependent manner, while propionate alone dose-dependently increased transepithelial electrical resistance in T84 and Caco-2 cells (Mariadason et al., 1997; Suzuki et al., 2008). Elamin et al. (2013) also reported that pretreatment of Caco-2 cells with 4 mmol/L propionate significantly alleviated the ethanol-induced barrier dysfunction, tight junction and F-actin disruption, and metabolic stress. Consistently, our results demonstrated that oral administration of sodium propionate could restore intestinal barrier function, at least in part, through inhibiting the down-regulation of tight junction and its associated protein in colitis mice. It is believed that sodium propionate cannot reach the colon due to its rapid gastric and duodenal absorption. Therefore, to verify whether oral sodium propionate could reach the colon, the concentration of sodium propionate in the caecum content needs further research.

Chronic inflammation as a hallmark of IBD often results from the recruitment and activation of immune cells from the circulation. It is postulated that intestinal tight junction barrier defects allow for paracellular permeation of noxious luminal antigens that induce inflammatory response. It was found that propionate decreased the generation of proinflammatory cytokines in a co-culture system combining Caco-2 cells with human whole blood (Hamer et al., 2008). Propionate could also diminish TNF-α production and release in neutrophils upon stimulation by lipopolysaccharide (Tedelind et al., 2007; Vinolo et al., 2011). These findings *in vitro* show that propionate has a favorable effect on IBD by attenuating activation of macrophages and neutrophils. The present study *in vivo* found that sodium propionate inhibited the up-regulation of proinflammatory factors IL-6, IL-1β, and TNF-α mRNA level in the colon of colitis mice.

Additionally, proinflammatory factors activate macrophages and neutrophils to infiltrate into the colonic mucosa, which in turn stimulates the production of reactive oxygen species (ROS), particularly superoxide, leading to oxidative stress (Roessner et al., 2008). ROS and reactive nitrogen species (RNS) produced by macrophages and neutrophils may further aggravate the inflammatory response and cause intestinal mucosal damage in IBD (Piechota-Polanczyk and Fichna, 2014). CD68 protein is known as a cell surface glycoprotein expressed in mature macrophages in the intestinal lamina propria and serves as a marker of macrophage and monocyte infiltration into the colon (Caprioli et al., 2013). We found that macrophages with CD68 marker were increased in colonic mucosa, and sodium propionate inhibited macrophage infiltration into the colonic mucosa in DSS-induced mice. We also found that sodium propionate decreased the MPO activity and increased the CAT and SOD activities in colon and serum. Taken together, our results indicate that sodium propionate alleviated inflammation and oxidative stress by inhibiting macrophage infiltration into the intestinal mucosa in DSS-induced colitis mice. However, how sodium propionate decreases macrophage infiltration into the intestinal mucosa and regulates macrophage function needs further investigation.

It was demonstrated that the level of activated STAT3 was higher in intestinal epithelial cells from patients with active ulcerative colitis compared with that in patients with inactive disease or healthy controls, and this level was positively correlated with the severity of colitis (Nguyen et al., 2015). We found that sodium propionate inhibited phosphorylation of STAT3 induced by colitis, which is consistent with the previous report that multi-fiber mix feeding decreased p-STAT3 expression in colonic mucosa of IL-10^-/-^ mice (Wang et al., 2016). STAT3 is a transcriptional activator and activated by a variety of cytokines, growth factors, and oxidative stress as well (Han and Theiss, 2014). But how sodium propionate inhibits STAT3 phosphorylation in colitis needs further study.

Moreover, no *in vivo* toxicity was observed after oral administration of 1% sodium propionate in this study, confirming the safety of sodium propionate. Although the colitis mice treated with propionate could not recover to normal, it is worth noting that low-dose (1%) oral sodium propionate attenuated acute DSS-induced colitis. It is important to avoid overdosing sodium propionate in clinical practice, as it may exacerbate rather than ameliorate colitis. Future studies are needed to compare the efficacy and safety of low-dose vs. high-dose sodium propionate oral therapy.

In summary, the present study demonstrated that oral administration of sodium propionate exerted beneficial effects on the intestinal epithelium by improving intestinal barrier function, inhibiting inflammation, and modulating oxidative stress through STAT3 signal pathway in DSS-induced colitis mice. Our study not only provides *in vivo* evidence for but gains preliminary mechanistic insights into the potential therapeutic benefits of sodium propionate for the management of colitis.

## Author Contributions

YW, L-cT, and Z-bW contributed to the conception and design of the study, and performed the research. W-yL and SS contributed to the acquisition, analysis and interpretation of data. L-cT and Z-bW drafted the manuscript. LL, L-cZ and D-fS supervised the project and revised the manuscript critically for important intellectual content. All authors have approved the final vision of this manuscript.

## Conflict of Interest Statement

The authors declare that the research was conducted in the absence of any commercial or financial relationships that could be construed as a potential conflict of interest.
